# Multiple myeloma with concurrent immune thrombocytopenic purpura

**DOI:** 10.3332/ecancer.2020.1012

**Published:** 2020-02-20

**Authors:** Humaira Sarfraz, Kartik Anand, Shujuan Liu, Shilpan Shah

**Affiliations:** 1 Department of Internal Medicine, Houston Methodist, Houston, TX 77030, USA; 2 Houston Methodist Cancer Center/Weill Cornell Medicine, Houston, TX 77030, USA; 3 Department of Pathology, Houston Methodist, Houston, TX 77030, USA; * Equal contribution

**Keywords:** multiple myeloma, ITP, thrombocytopenia, auto immune disorder, immunoglobulin

## Abstract

Multiple myeloma (MM) is the second most common haematological malignancy in the USA. MM has been linked to various autoimmune disorders in many studies; one systemic review even suggested an increased risk of MM among patients with autoimmune disorders. MM is associated with many haematological, rheumatologic and neurological conditions. A few case reports suggest that MM can be associated with immune thrombocytopenic purpura (ITP), although this is rare. We present a case of MM with concurrent ITP which was refractory of steroids and intravenous immunoglobulin but had a response with anti-neoplastic therapy for MM. We also review all the cases of ITP with MM described in the literature. If conventional treatment for ITP associated with MM fails to improve platelet count, anti-neoplastic therapy for MM should be considered.

## Introduction

Multiple myeloma (MM) is a plasma cell disorder characterised by clonal proliferation of malignant cells in the bone marrow with monoclonal protein in the serum and/or urine and is associated with end-organ damage including anaemia, hypercalcemia, renal dysfunction and bone disease [1]. MM is the second most common haematological malignancy with an incidence of 6.2/100,000 [2]. MM at presentation can be symptomatic or asymptomatic; for diagnosis, at least >10% clonal bone marrow plasma cells plus ≥1 multiple myeloma defining events are required [3]. Aetiology of MM remains unknown; some of the risk factors are advanced age, family history, male gender and environmental factors [4]. Several studies suggest a link between autoimmune disorders and MM, one systemic review suggested patients with autoimmune disease are at high risk for development of MM [5]. MM is associated with many haematological, rheumatologic and neurological conditions [6]. A few case reports suggest that rarely MM can be associated with immune thrombocytopenic purpura (ITP) [7]. The likely mechanisms, which may explain the correlation between MM and ITP, include that autoimmune conditions may lead to chronic B cell activation and development of MM. However, MM itself is also associated with the development of autoimmune phenomena. Third, thrombocytopenia may occur as a result of certain drugs used for treatment of MM. Most cases of ITP associated with MM respond to steroids and intravenous immunoglobulins (IVIg) [7]. Here, we present a case of MM with concurrent ITP which was refractory to corticosteroids and IVIg and had response to anti-neoplastic therapy for MM.

## Case presentation

**Figure 1.**
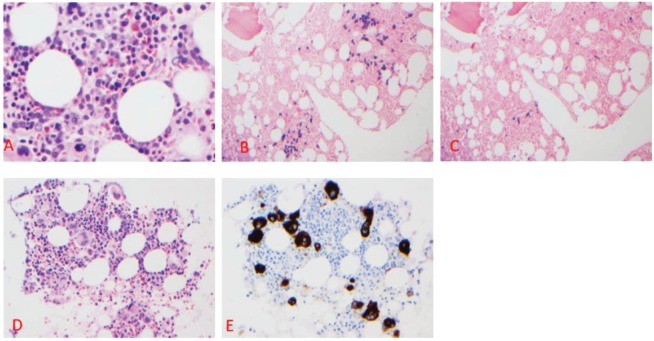
Clustering of lambda-light chain restricted plasma cells on Bone marrow biopsy section (A–C). A. HE × 200; B. Lambda light chain ISH x100; C. Lambda light chain ISH x100. D. Focal loose clustering of megakaryocytes. A HE ×200; E CD61 immunohistochemical stain ×200.

A 60-year-old male with a past medical history of bipolar disorder and schizophrenia on olanzapine presented to the hospital with generalised weakness and weight loss for the past 2 months. His physical exam was unremarkable for any petechiae, bleeding, bruises, hepatosplenomegaly or evidence of lymphadenopathy. Laboratory examination showed haemoglobin of 5.9 g/dL, WBC 2.88 k/μL with normal differential, platelet count of 1 k/μL, creatinine 1.1 mg/dL, calcium 8.6 mg/dL, albumin 2.7 g/dL and total protein 7.6 g/dL. Prothrombin was within normal limits (WNL), and activated partial thromboplastin time (PTT) was elevated at 42.3 seconds. Further work regarding the elevation of PTT revealed the presence of lupus anticoagulant. The D-dimer level was elevated at 2.9 μg/mL, and fibrinogen was elevated as well at 463 mg/dL. Haptoglobin was WNL at 189 mg/dL, LDH was elevated at 506 U/L and reticulocyte index was low at 2 with no spherocytosis or schistocytes on peripheral smear exam which ruled out haemolysis. Immunoglobin quantification showed increased IgG at 2,157 mg/dL, IgA WNL at 338 mg/dL and decreased IgM at <25 mg/dL. Serum and urine protein electrophoresis with immunofixation showed the presence of IgG lambda paraproteinemia at a concentration of 0.8 and 0.6 g/dL, respectively. ESR was found to be elevated at 55 mm/1st hour and beta-2 microglobulin was elevated at 4.1 mg/L. The patient was transfused packed red blood cells with an appropriate rise in haemoglobin, but there was no rise in platelet count checked 1 hour after platelet transfusion indicating peripheral destruction. Bone marrow biopsy was performed which showed normocellular marrow with 30% lambda restricted plasma cells (Figure 1A–C) and focal megakaryocytic hyperplasia, staining positive by CD61 (Figure 1D–E). Congo red staining for amyloid was negative. Skeletal survey showed no lytic lesions. Work up done to rule out secondary causes of thrombocytopenia, including HIV, hepatitis C virus, rheumatological workup with anti-nuclear antibody and imaging to detect splenomegaly, was unremarkable. Hence, with all the work described earlier, a diagnosis of concurrent ITP and MM was established. This patient satisfied the criteria for MM due to the presence of >10% clonal plasma cells in the bone marrow along with anaemia [3]. An initial trial for ITP treatment with IVIg 1 g/kg for 2 days and dexamethasone 40 mg daily for 4 days did not lead to an increase in platelet count. Given his refractory thrombocytopenia and concurrent multiple myeloma, he was subsequently started on cyclophosphamide, bortezomib, dexamethasone (CyBorD). On day 6 of cycle 1, a rise in platelet count to 29 k/μL was noted. Subsequent days thereafter, the platelet count continued to increase and peaked at 171 k/μL before the start of his next cycle of chemotherapy. From a multiple myeloma standpoint, there was a decrease in paraprotein load from 0.8 to 0.3 g/dL. He recently received his cycle 2 of induction chemotherapy with a future plan to repeat a bone marrow biopsy and move on to high dose chemotherapy followed by stem cell rescue.

## Discussion

Here, we describe a case of concurrent immune thrombocytopenic purpura with multiple myeloma which was refractory to treatment with pulse dexamethasone and IVIg but responded to treatment with chemotherapy for Multiple Myeloma with CyBorD. While thrombocytopenia may be seen with multiple myeloma commonly, ITP has rarely been encountered as an association with multiple myeloma. In most cases, thrombocytopenia results from chemotherapy during multiple myeloma treatment or marrow infiltration by the plasma cells. In our case, the absence of an increase in platelet count with platelet transfusion indicated a destructive aetiology for thrombocytopenia. Moreover, the absence of secondary causes and megakaryocytes noted on bone marrow exam confirmed diagnosis of ITP. To date, 11 cases of ITP associated with MM have been reported (Table 1), comprising of 7 males and 4 women. M proteins in all patients were of IgG type. Four cases (1, 6, 9, 10) were diagnosed with ITP after receiving chemotherapy for MM, while three cases of ITP (4, 5, 11) were diagnosed before MM. As per the literature review, there are only three cases (3, 7, 8) of Multiple Myeloma and ITP being diagnosed concurrently (2, 4, 5). The age spectrum of the previous cases described range from 49 to 78 years. In the case series by Gupta *et al* ], their patient with concurrent ITP and MM, thrombocytopenia responded to treatment with IVIg and was subsequently started on chemotherapy with VAD for MM treatment. In the case report by Tabata *et al* [9], initially, the patient was diagnosed with MM with mild thrombocytopenia noted with increased megakaryocytes in the bone marrow. He was treated for MM with melphalan and prednisone but could not receive subsequent rounds of chemotherapy due to severe neutropenic infections. It was observed that with the progression of MM, platelet count continued to decrease while the megakaryocyte concentration was preserved indicating that there could potentially be an association between MM severity and thrombocytopenia which was demonstrated by the rise in platelet associated antibody. The platelet count in that case responded to cepharanthine (natural alkaloid) most likely associated with decreased cytokine production. In the case described by Yao et al, immunosuppression for ITP was linked to MM. For cases in which MM was diagnosed before ITP (1, 6, 9, 10), it is likely that MM led to certain autoimmune sequelae resulting ITP. In such cases, medication induced effects would also need to be considered. On the other hand, when MM follows ITP diagnosis (4, 5, 11), most likely mechanism implicated is chronic B-cell activation. While these cases may allude to the cause of an association between MM and ITP, it is still difficult to ascertain the exact mechanism given paucity of evidence and heterogeneity of disease.

In our case, there was a lack of response to IVIg and dexamethasone to ITP but there was a remarkable response of thrombocytopenia to treatment with CyBorD. While bortezomib has been found to have a good response in MM, bortezomib itself is implicated in causing thrombocytopenia in some cases too. The rise in platelets seen in our patient with concurrent ITP raises the possibility that manifestation of ITP is perhaps caused by MM itself and the treatment of MM reciprocally leads to its resolution [10, 11].

The pathogenesis of ITP with MM is poorly understood. It has been hypothesised that immune alterations promote the generation of autoimmune platelet antibodies [8]. This case presentation describes a rare autoimmune manifestation of MM (ITP) and highlights that the treatment of MM with combination chemotherapy can lead to resolution of ITP refractory to IVIg and corticosteroids treatment.

## Conclusion

In conclusion, we discuss a rare case of MM complicated by concurrent ITP. In contrast to most common associations of ITP and MM which usually respond to IVIg, steroids or splenectomy, ours was a unique case where administration of chemotherapy (CyBorD) resulted in normalisation of platelet count. This response suggests the implication of abnormal immune mechanisms in ITP associated with MM.

## Conflicts of interest

None.

## Funding

The authors received no specific funding for this work.

## Figures and Tables

**Table 1.** Cases of ITP with MM described in the literature till date excluding the current case.

Case	Reference	Age/Gender	M protein type at diagnosis	M protein conc (g/dL)	Platelet 10^9^/L	ITP Diagnosis timing	ITP Management	Alive/Cause of death
1	Verdirame *et al* [12]	67 y/F	IgG/lambda	2.4	10	After MM Tx	Prednisone, splenectomy	Alive
2	Verdirame *et al* [12]	55 y/M	IgG/kappa	3.4	48	During MM Tx	Prednisone, splenectomy	Alive
3	Gupta *et al* [8]	49 y/M	IgG/lambda	4.0	21	At MM Dx	IVIg, VAD	Sepsis
4	Gupta *et al* [8]	36 y/M	IgG/kappa	11.9	5	Before MM Dx	Prednisone, IVIg, splenectomy, VAD	Sepsis
5	Gupta *et al* [8]	45 y/M	IgG/lambda	6.1	20	Before MM Dx	Steroid, IVIg, splenectomy	Alive
6	Falco *et al* [13]	45 y/M	IgG/kappa	1.8	<10	After MM Tx	Prednisone, IVIg	N/A
7	Siniscalchi *et al* [14]	67 y/F	IgG/kappa	2.6	3	At MM Dx	Prednisone, IVIg, Rituximab	Alive
8	Tabata *et al* [9]	78 y/M	IgG/kappa	4.18	17	At MM Dx	Melphalan, Prednisone, Cepharanthine	Unknown cause of death
9	Faller *et al* [7]	66 y/M	IgG/kappa	1.00	<10	After MM Tx	IVIg	Alive
10	Faller *et al* [7]	41 y/F	IgA/kappa	N/A	<10	After MM Tx	IVIg, Dexamethasone	GI bleed
11	Yao *et al* [15]	61 y/M	IgG/kappa	N/A	27	Before MM Dx	Refractory ITP; Prednisone, IVIg, Danazol, IFN, AZT	Alive
